# Theranostic nanobubble encapsulating a plasmon-enhanced upconversion hybrid nanosystem for cancer therapy

**DOI:** 10.7150/thno.38684

**Published:** 2020-01-01

**Authors:** Wen-Tse Huang, Ming-Hsien Chan, Xueyuan Chen, Michael Hsiao, Ru-Shi Liu

**Affiliations:** 1 Department of Chemistry, National Taiwan University, Taipei 106 Taiwan;; 2 CAS Key Laboratory of Design and Assembly of Functional Nanostructures, and Fujian Key Laboratory of Nanomaterials, Fujian Institute of Research on the Structure of Matter, Chinese Academy of Sciences, Fuzhou, Fujian 350002, China;; 3 Genomics Research Center, Academia Sinica, Taipei 115 Taiwan;; 4 Department of Biochemistry College of Medicine, Kaohsiung Medical University, Kaohsiung, 807 Taiwan.; 5 Department of Mechanical Engineering and Graduate, Institute of Manufacturing Technology, National Taipei University of Technology, Taipei, 106 Taiwan

**Keywords:** near-infrared, plasmonic enhancement, upconversion hybrid nanosystem, dual-model phototherapy, cancer theranostics

## Abstract

Nanobubble (NB), which simultaneously enhances ultrasound (US) images and access therapeutic platforms, is required for future cancer treatment.

**Methods:** We designed a theranostic agent for novel cancer treatment by using an NB-encapsulated hybrid nanosystem that can be monitored by US and fluorescent imaging and activated by near-infrared (NIR) light. The nanosystem was transported to the tumor through the enhanced permeability and retention effect. The hybrid nanosystem comprised upconversion nanoparticle (UCNP) and mesoporous silica-coated gold nanorod (AuNR@mS) with the photosensitizer merocyanine 540 to realize dual phototherapy.

**Results:** With the NIR light-triggered, the luminous intensity of the UCNP was enhanced by doping holmium ion and emitted visible green and red lights at 540 and 660 nm. The high optical density state between the UCNP and AuNR@mS can induce plasmonic enhancement to improve the photothermal and photodynamic effects, resulting in cell death by apoptosis. The nanosystem showed excellent stability to avoid the aggregation of nanoparticles during the treatment. JC-1 dye was used as an indicator of mitochondrial membrane potential to identify the mechanism of cell death. The results of *in vitro* and *in vivo* analyses confirmed the curative effect of improved dual phototherapy.

**Conclusion:** We developed and showed the therapeutic functions of a novel nanosystem with the combination of multiple theranostic nanoplatforms that can be triggered and activated by 808 nm NIR laser and US.

## Introduction

Given the increasing popularity of nanotechnology in recent years, high-resolution imaging and low-harm treatment are expected for cancer diagnosis and therapy 1. The fluorescence of optical imaging is extensively used in nanomedicine for bioimaging 2. Conventional fluorescent materials possess a down-converted property, indicating that visible light is emitted through the absorption of a high-energy light source, such as ultraviolet (UV) light. The common problem in using UV light is a severely damaged biological tissue caused by high energy and short wavelength 3. The near-infrared (NIR) light window indicates that biological tissue has low absorbance in the NIR region, thereby allowing light penetration deeper than UV light 4. The NIR laser has many advantages, such as high spatial resolution, narrow emission band, and low biotoxicity 5. Upconversion nanoparticle (UCNP) exhibits the highest potential when the NIR laser is collocated with nanoparticles on the theranostics 6-8. The specific optical characteristics of UCNP cause energy conversion emitting short-wavelength energy (visible light) through the absorption of long-wavelength excitation (e.g., 980 nm or 808 nm laser) 2. Compared with the traditional treatment, NIR-triggered phototherapy produces a trend with non-invasion that can be classified into photothermal therapy (PTT) 9-11 and photodynamic therapy (PDT) 12-16. The nanocomposite is more useful than the single material because the former can be tuned flexibly with multiple applications and different purposes, thereby creating a complete theranostic nanoplatform 17-21. Gold nanorod (AuNR) is commonly used for PTT and show good thermal generation via luminous energy 22.

The novel nanocomposite of AuNR and UCNP with photosensitizer loading shows better therapeutic efficacy of dual phototherapy (PTT and PDT) than single therapy 23-26. Multimodal imaging and theranostics also exhibit significant therapeutic effects via different nanocomposite systems 27-33. Abundant energy from UCNP is needed to advance the photothermal effect of AuNR 34. Adding holmium ions (Ho^3+^) makes plasmonic enhancement strengthen the red emission intensity of UCNP 35. The plasmon-enhanced reactive oxygen species (ROS) production of PDT can be achieved through the optimization of photosensitizer loading amount and efficient distance with tuning the silica shell thickness. On the basis of localized plasmon surface resonance, the upconversion luminescence of UCNP can be improved with AuNR by further increasing the ROS content 12. Furthermore, treatment with nanomaterials faces critical challenges in stability and accuracy 36. Before accumulating in the tumor, nanocomposites could react with proteins or organelles and even decompose, causing biotoxicity. Therefore, a good nanocarrier, such as nanobubble (NB), is needed to solve the aforementioned problems 37. NB, a type of contrast agent, are used in ultrasound (US) imaging to overcome low resolution and are suitable for diagnostic applications because of the differentiation between biological tissues via alterations in image contrast 38. The size of bubbles is determined by the ratio of phospholipids and surfactants. Doping of small lipids or increasing amount of polyethylene glycol (PEG) surfactant reduces the size and distribution of bubbles 39. Compared with other contrast agents, such as microbubble (MB) or micelle 40, NB can be accumulated passively and modified easily to transport drugs with a size of 100-500 nm 41,42. NB can exploit the enhanced permeability and retention (EPR) effect 43, which renders NB more suitable than MB for clinical application 1,44. Angiogenesis is the uncontrolled formation of new capillaries from pre-existing ones stimulated by tumor angiogenic factors. Tumor vasculature-associated endothelial cells are characterized by large gaps (200-1200 nm) caused by angiogenesis 45. NB passively target tumor through EPR, and US imaging has confirmed that this effect is only exploited by particles at the nanoscale or smaller. Apoptosis is important for the development of new drugs and cancer treatment 46. The mitochondrion is an organelle where several crucial processes occur, such as caspase activators releasing cytochrome c, the difference in electron transportation, and change in the mitochondrial transmembrane potential (ΔΨm) 47,48. JC-1 dye is a good indicator that shows different colors from green to red in different membrane potentials, thereby possibly reflecting the health of the mitochondrion 49,50.

In the present study, we developed a novel nanosystem that shows significantly enhanced US imaging with optimal luminescence efficiency and stimulated a conducive, synergistic phototherapeutic effect. The theranostic agent was an AuNR@UCNP conjugate encapsulated inside an NB (AuNR@UCNP@NB). An energy of 808 nm NIR light passed from the UCNP to the AuNR, thereby conferring the AuNR a plasmonic effect with high temperature. Moreover, the plasmonic enhancement caused a synergistic effect on the merocyanine 540 (MC540) photosensitizer to produce ROS, thereby activating the apoptotic pathway of cancer cells. Our nanosystem can be activated by 808 nm laser to prevent water absorption with deep tissue imaging and improve the therapeutic effect of PTT and PDT with an NB nanocarrier. Our newly developed dual-functional nanosystem can be utilized to improve NIR phototherapy with multiple imaging capabilities for successful cancer treatment.

## Methods

### Synthesis of mesoporous silica-coated AuNR with the loading photosensitizer MC540 (AuNR@mS)

AuNR was synthesized using the modified seed-mediated growth method. The seed solution was prepared using CTAB (0.2 M, 5 mL) and HAuCl_4_ (0.1 M, 25 µL) solutions that were reduced to Au seeds by adding NaBH_4_ (0.01 M, 0.6 mL) in an ultrasonic environment with pipetting. The growth solution was prepared from the CTAB solution (0.2 M, 125 mL) with sodium salicylate (0.8 g) at 55 °C and mixed with the AgNO_3_ (4 mM, 6 mL) and HAuCl_4_ (1 mM, 250 mL) solutions at 30 °C for 15 min. Thereafter, the ascorbic acid solution (0.064 M, 1 mL) was added without disturbance. After the addition of the seed solution (0.8 mL) into the growth solution, the stirring was turned off, and the solution was incubated at 30 °C overnight. The AuNR was purified by centrifugation at 10,000 rpm for 30 min. TEOS (30 μL) in the AuNR solution (10 mL) was added in an alkaline environment with constant stirring for 2 days. The solution was refluxed with HCl at 60 °C for 6 h to form the mesoporous silica-coated AuNR (AuNR@mS). AuNR@mS was purified by centrifugation at 10,000 rpm for 20 min and redispersed in deionized water.

### Synthesis of NaYF_4_:Yb/Er/Ho@NaYF_4_:Yb/Nd@NaYF_4_ three layers UCNP (UCNP)

UCNP was synthesized by high-temperature coprecipitation. The core of the NaYF_4_:Yb/Er/Ho nanocrystal was prepared by adding 0.64 mmol Y(CH_3_CO_2_)_3_, 0.144 mmol Yb(CH_3_CO_2_)_3_, 0.004 mmol Er(CH_3_CO_2_)_3_, and 0.012 mmol Ho(CH_3_CO2)_3_ with 6 mL of OA and 14 mL of ODE, and it was heated to 120 °C to evaporate the water. Thereafter, this mixture was heated to 170 °C to allow ligand formation between the lanthanide ions (Ho, Er, and Yb) and OA. The precursor was cooled to 45 °C, and then the methanol solution with 2 mmol NaOH and 3.16 mmol NH_4_F was added and stirred for 1.5 h. The temperature was increased to 120 °C to remove the methanol for 10 min under vacuum and heated at 305 °C for 1.5 h in the nitrogen environment. The NaYF_4_:Yb/Er/Ho nanocrystal was purified by centrifugation with anhydrous ethanol and then stored in cyclohexane. The NaYF_4_:Yb/Nd shell was synthesized by repeating the core formation process but using different precursor concentrations, namely, 0.51 mmol Y(CH_3_CO_2_)_3_, 0.034 mmol Yb(CH_3_CO_2_)_3_, and 0.136 mmol Nd(CH_3_CO_2_)_3_. At the shell layer-forming steps, the NaYF_4_:Yb/Er/Ho core nanocrystal was prepared and added with the host materials of the shell into the precursor. The shell-forming and precipitation steps were repeated to form the NaYF_4_:Yb/Er/Ho@NaYF_4_:Yb/Nd core-shell UCNP. The synthesis was repeated thrice to form the core-shell-shell UCNP. The outer shell was composed of NaYF_4_. Therefore, the precursor contained only Y(CH_3_CO_2_)_3_. The oil phase sample was washed with alcohol-diluted HCl to remove the OA and ODE ligands and obtain the water-soluble UCNP.

### Synthesis of NB

Three phospholipids were used to synthesize NB: DPPC, DPPA, and DSPE-PEG 2000. The phospholipids were dissolved in chloroform (4 mL), and a phospholipid film formed after natural evaporation. The surfactant with glycerol (10 %) and Pluronic F-127 (8 mg) was added to the phospholipid film and placed in a shaking incubator for 1 h at 37 °C. The solution was sonicated for 2 min to agitate the particles and form the C_3_F_8_-cored NB under N2 and C_3_F_8_ gases, which were stored at 4 °C.

### Modification of AuNR@mS with 808 nm UCNP nanocomposite (AuNR@UCNP)

The nanocomposite composed of AuNR@mS and 808 nm UCNP was conjugated via classical electrostatic interaction. The UCNP solution (2 mg/mL) mixed with the protamine solution (20 mg/mL) was stirred for 24 h under room temperature. The coated UCNP was purified by centrifugation at 7600 rpm for 6 min. Thereafter, the coated UCNP solution was conjugated with the AuNR@mS solution in different ratios and stirred for 1 h, which was purified by centrifugation thrice at 7600 rpm for 6 min.

### Synthesis of AuNR@UCNP embedded in the NB nanosystem (AuNR@UCNP@NB)

The synthesis of AuNR@UCNP@NB was similar to that of the NB. When the thin phospholipid film was formed, the surfactant-containing glycerol and AuNR@UCNP were simultaneously mixed with the thin film. This process was performed in a shaking incubator at 37 °C for 1 h and sonicated for 2 min to form AuNR@UCNP@NB. The samples were stored at 4 °C.

### Upconversion luminescence lifetime measurement

The luminescent lifetimes of the UCNP and AuNR@UCNP were determined with a spectrometer (FSP920-C, Edinburgh Instrument) customized UV to mid-infrared steady-state and phosphorescence lifetime. The excitation source was supplied from the tunable mid-band optical parametric oscillator pulse laser (410-2400 nm, 10 Hz, pulse width around 5 ns, Vibrant 355II, OPOTEK).

### Phototherapy experiment

The two parts of the light treatment are as follows. The first part is the PTT analysis, in which different ratios of the AuNR@UCNP at 1 mg/mL were added to a cuvette tube and continuously stirred by magnetic force. The solution was irradiated with an 808 nm laser at 1.5 W/cm^2^ for 60 min, while the temperature of the solution was measured with a thermocouple at 20 s intervals. The second part is the PDT analysis, in which ABDA was reacted with ^1^O_2_ to detect ROS in the aqueous solution. AuNR@UCNP with different AuNR ratios were dispersed in 1 mg/mL deionized water. The solution was placed in the cuvette and then stirred. Thereafter, the solution was irradiated with 1.5 W/cm^2^ 808 nm laser for 60 min. The ROS production was confirmed by detecting the absorption of ABDA at 378 nm. The absorbance spectra of the mixture after the NIR laser irradiation were recorded using Fluoromax-3 (Horiba, Japan). After NIR laser irradiation, the absorption spectra of the mixture were recorded using a UV-1700 spectrophotometer (Shimadzu, Japan).

### Cellular uptake and localization of AuNR@UCNP@NB

Approximately 20,000 cells of Beas2B and A549 were suspended in 1 mL of medium and planted on a 24-well plate slide for 12 h. A 250 μg/mL nanoparticle composite was added to the culture at 37 °C with 5% carbon dioxide for 12 h. The cells were washed with phosphate buffer solution (PBS, 10 mM, pH 7.4) and then fixed in 4% paraformaldehyde fixative (paraformaldehyde) to maintain their intrinsic form. Subsequently, the nuclear stain DAPI was added for nuclear staining. After 5 min of incubation, the dye was removed and observed by laser scanning confocal microscopy (LSCM). The nucleus was excited by a 408 nm UV laser, while the emission image was detected at 450-500 nm. The nanocomposite was excited by a NIR laser at 808 nm, and its emission was detected at 630-680 nm.

### *In vitro* cell viability and cytotoxicity of AuNR@UCNP@NB

The Beas2B normal lung and A549 lung cancer cell lines were selected as the observation objects of material biocompatibility. The KSFM medium was added with pituitary fluid and epidermal growth factor to maintain the growth of the Beas2B cells. By contrast, the F12K medium with 1% PSG was supplemented with 10% fetal bovine serum as the culture solution for A549 cells. Both of these cell lines were cultured at 37 °C and 5% carbon dioxide. Approximately 2000 cell lines were cultured in 96-well plates for 12 h, whereas AuNR@UCNP and AuNR@UCNP@NB at 3, 9, 27, 81, and 250 μg/mL were added to the individual wells for 48 h. The cell dye Alamar Blue was likewise added. Cell staining was performed, and the fluorescence intensity of Alamar Blue was detected by a fluorophore to count cell viability. The preceding steps were repeated and exposed to 808 nm laser to determine cell cytotoxicity.

### ROS evaluation and apoptosis mechanism of PDT

The condition for the detection of 100 μM DCFH dye was the same as that for the samples that were tested by ABDA dye. This dye reacted irreversibly with ^1^O_2_ to generate DCF molecular in the cytosol, and the green fluorescence of the DCF emitted at approximately 550 nm was detected by LSCM. The JC-1 mitochondrial dye was used to detect the depolarization of the mitochondria to observe the changes in the mitochondrial membrane potential in living cells and observed by LSCM. The color change of the fluorescence was used as a basis to investigate the mechanism of apoptosis induced by light-sensing substances. JC-1 is a positively charged dye that is easily attracted by the negative electricity on the mitochondrial membrane and aggregates in the inner membrane of the mitochondria. The red fluorescence of the wavelength indicates a healthy cell and can be excited under 610 nm laser. A drop in the membrane potential due to the depolarization of the mitochondria indicates the early stage of apoptosis, which was dispersed in the form of a monomer and exhibited green fluorescence at 570 nm.

### Flow cytometric analysis of apoptosis

Apoptotic cells were identified by using the JC-1 Kit in accordance with the manufacturer's protocol. Beas2B normal lung and A549 lung cancer cells (2 × 10^5^ cells) were treated with AuNR@UCNP, UCNP + MC540, and AuNR@UCNP@NB (100 μg/mL) and exposed to 808 nm laser for 72 h. The cells were detached by trypsin-EDTA, washed with PBS, and then stained with 5 μL of JC-1 dye for 15 min in the dark. Apoptosis was analyzed via FAC Scanto flow cytometry. The results were investigated using CellQuest software.

### Western blot analysis

The cells treated with NB, UCNP, AuNR@UCNP, UCNP + MC540, and AuNR@UCNP@NB were examined through Western blot. Equal quantities of protein (20 μg) were separated using SDS-PAGE and transferred onto nitrocellulose membranes (Amersham Bioscience, Buckinghamshire, UK) through a Bio-Rad wet transfer system. After blocking with 5% non-fat dried milk in PBS/Tween20 buffer for 1 h at room temperature, the membranes were incubated with caspase-3, PARP, and α-tubulin antibody for 1 h at room temperature. Thereafter, the membranes were incubated with horseradish peroxidase-conjugated secondary antibody for 1 h at room temperature. Proteins were visualized using enhanced chemiluminescence.

### *In vivo* phototherapeutic effect

Animal experiments have been approved by the Institutional Animal Care and Utilization Committees of Academia Sinica (IACUC NO. 16-05-957). The A549 lung cancer cell line (5 × 10^6^ cells/100 µL) was injected subcutaneously on the right thigh of the mouse. The NB and AuNR@UCNP@NB nanocomposite (100 mg) was intratumorally injected into the tumors when they had grown to 21 mm^3^. After 12 h incubation, irradiation with 500 mW/cm^2^ of 808 nm NIR laser was performed for 30 min with 5 min intervals of cooling down and irradiation in the fourth and fifth weeks.

### Histochemical staining (hematoxylin and eosin (H&E) staining/immunohistochemical staining)

Thereafter, all tumors were acquired after the fifth week. The tumor sections were formalin-fixed and paraffin-embedded. Cross-sections of the tumor were stained using H&E and immunohistochemistry (IHC). Each tumor that was taken from the formalin-fixed paraffin-embedded tissues was selected by morphology typical of the diagnosis. H&E staining assisted by Leica ST5010 Autostainer XL and IHC staining was performed on serial tissue sections cut from the tissue microarray using an automated immunostainer. In brief, the sections were first dewaxed in a 60 °C oven, deparaffinized in xylene, and rehydrated in graded alcohol. Antigens were retrieved using heat-induced antigen for 30 min in DAKO retrieval solution (pH 6.1). Slides were blocked by H_2_O_2_ and stained with polyclonal rabbit anti-human Ki67 antibody and TUNEL reagent overnight. The sections were subsequently counterstained with a second antibody, ABC kit, and mounted to obtain the final IHC stained slides. All the stained tumor sections were observed by a Leica Aperio AT2 scanner.

## Results and Discussion

**Figure 1:**
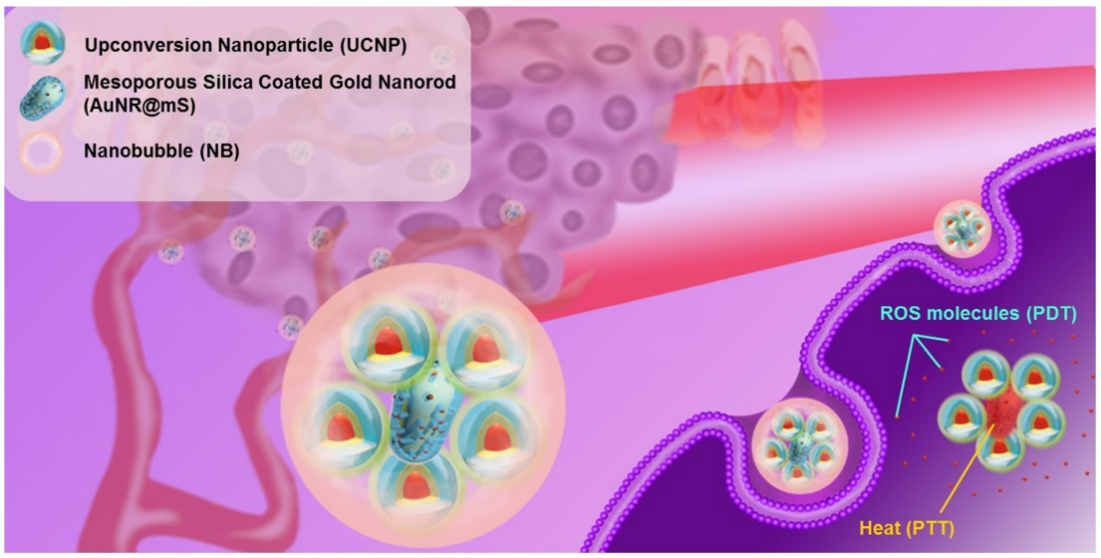
Schematic of the upconversion hybrid nanosystem designed in the NIR-triggered phototherapy.

An upconversion hybrid nanosystem with dual phototherapy (PTT and PDT) was designed in this research (Figure 1). The nanosystem triggered from 808 nm laser and US was composed of the nanocomposite AuNR@UCNP with the nanocarrier NB. We conjugated the controlled AuNR with UCNP to strengthen the photothermal effect via a plasmonic enhancement to obtain the improved curative effect of phototherapy. The photosensitizer MC540 was loaded in the mesopores with AuNR@mS, which can produce ROS. The therapeutic effect of the nanosystem was substantial in the *in vitro* and *in vivo* tests.

### Characterization of AuNR@mS and UCNP

Each nanomaterial was identified to confirm the physical and optical characterization. First, the MC540-loaded AuNR@mS was synthesized as the major curative source of dual phototherapy (Figure S1A). The good dispersity of AuNR and AuNR@mS in deionized water was observed using transmission electron microscopy (TEM) with an aspect ratio of 2.7 (Figure S1B) and silica thickness of 9 nm (Figure S1C). The two characteristic absorption peaks from MC540 and AuNR@mS can absorb the 530 nm and 650 nm fluorescence, respectively, emitted by UCNP. The strong absorbance enhancement between 490 nm and 540 nm confirmed the successful loading of MC540 (Figure S1D). MC540 was loaded in AuNR@mS via mixing two solutions, thereby showing the 42 nm hydrodynamic diameter (Figure S1E). After four times centrifugation, the unloaded or superficial adsorption of MC540 was washed with water. The real loading amount in the mesopores was calculated to be approximately 23% (Figure S1F). Second, the core-shell-shell (three layers) structure of the UCNP synthesized by co-precipitation was developed in the previous research, in which the third layer structure could protect the crystal damage of UCNP 23. X-ray diffraction patterns showed that the β-NaYF_4_ hexagonal phase (JCPDS 16-0334) of the UCNP was maintained after the core-shell-shell synthesis (Figure S2A). The hydrodynamic diameter of the UCNP was approximately 27 nm in the water solution (Figure S2B). However, the absorbance spectrum showed small peaks around 808 nm, thereby confirming that the UCNP can be triggered by 808 nm laser (Figure S2C). After the improvement, the erbium and holmium ions of the luminescence center caused double energy transition with three characteristic peaks around 420 nm (^5^G_5_ → ^5^I_8_ and ^2^H_9/2_ → ^4^I_15/2_), 540 nm (^5^S_2_ → ^5^I_8_ and ^4^S_3/2_ → ^4^I_15/2_), and 660 nm (^5^F_5_ → ^5^I_8_ and ^4^F_9/2_ → ^4^I_15/2_), as shown in the photoluminescence (PL) spectrum. To approach the improved photothermal effect of AuNR@mS, the luminescent intensity of the UCNP at 660 nm was enhanced by 36% by doping Ho^3+^ at the core of the nanocrystal (Figure S2D).

### Characterization of AuNR@UCNP

**Figure 2:**
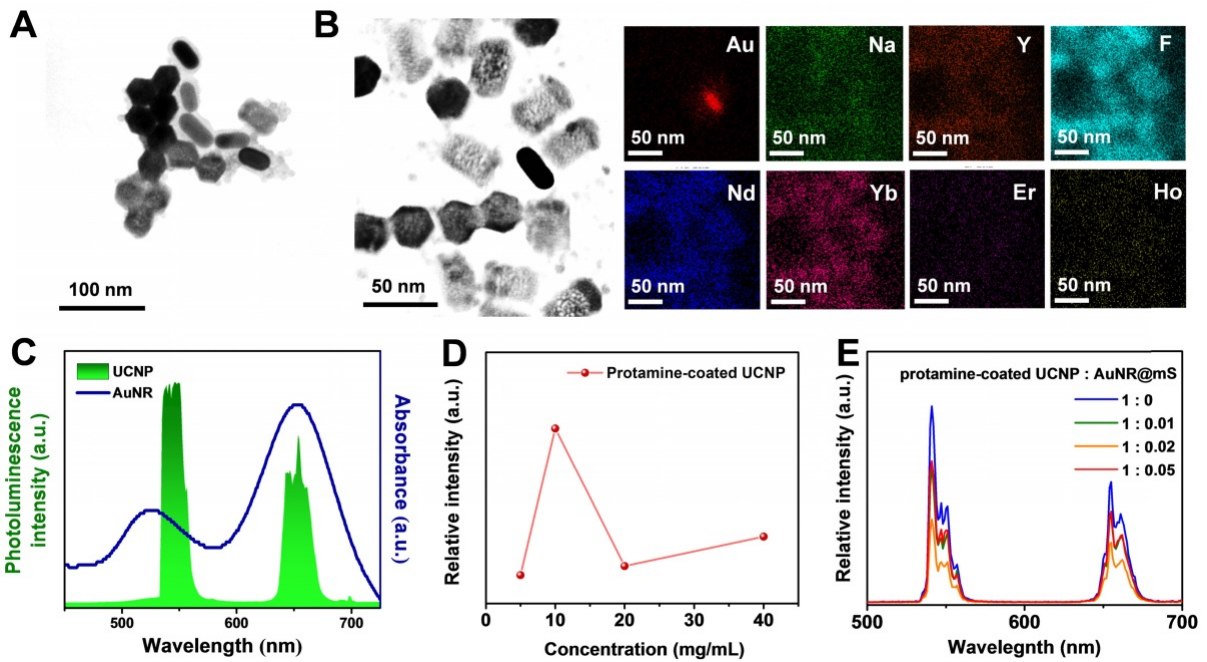
** (A)** Morphology of AuNR@UCNP with TEM image and **(B)** EDS element mapping. **(C)** Absorbance spectrum of AuNR with PL spectrum of UCNP. Photoluminescence intensity of UCNP in different concentrations of **(D)** protamine modification and **(E)** AuNR@mS combination.

For improved energy transfer and use in the biomedical field, the UCNP was changed to the water phase via pickling and combined with AuNR@mS through electrostatic adsorption. Thus, the protamine molecule with positive polarity was chosen as the link between the UCNP and AuNR@mS 51. Protamine is a Food and Drug Administration-approved molecule that can provide positive effects on the UCNP surface because of its low biotoxicity. The protamine layer of the UCNP can be distinguished clearly from the TEM images with 2 nm thickness (Figure S3A). In the Fourier-transform infrared spectrum, the carbon chain of the hydrophobic ligands at 2923, 2854 cm^-1^ shifted to the hydroxide of the hydrophilic ligands, whereas the characteristic peaks of protamine were also observed after surface modification (Figure S3B). The protamine-coated UCNP was conjugated with AuNR@mS via electrostatic adsorption to generate the upconversion hybrid nanocomposite (AuNR@UCNP) (Figure 2A). The high-concentration TEM sample showed that the dispersion was low. The exact components of the AuNR@UCNP were confirmed by EDS mapping in Figure 2B. The results of zeta potential indicated that the surface charge of AuNR was about 28.3 mV. After cladding the silica layer, the surface charge became negative approximately -21.2 mV. Cladding the protamine out of the UCNP changed the surface charge from 0.22 mV to 22.5 mV. The entirety of the AuNR@UCNP surface charge performed minimal positivity with 6.13 mV and the hydrodynamic diameter of 50 nm after the conjugation (Figure S3C). The 808 nm laser-triggered nanocomposite caused energy transfer from the UCNP to the AuNR, which was confirmed with the absorbance and PL spectrum in Figure 2C. The amount of protamine coated on the UCNP was optimized before conjugation. Different concentrations were coated on UCNP with 5, 10, 20, 40 mg/mL protamine solution. The highest emission intensity was detected when the 10 mg/mL protamine coated with UCNP was used (Figure 2D). Given that AuNR@mS absorbs most of the light energy from the UCNP, the ratio of AuNR@mS and protamine-coated UCNP was optimized to obtain the best energy transfer for dual phototherapy. The emission intensities of 540 nm (green light) and 660 nm (red light) declined considerably because of the absorption by AuNR and MC540 (Figure 2E). The change in a luminescent lifetime was observed after the conjugation of AuNR@mS to identify the efficiency of energy transfer. The luminescent lifetime of 654 nm was changed from 0.65 ms to 0.61 ms under 808 nm laser, thereby demonstrating the small decrease that the mechanism of radiative energy transfers instead of non-radiative of fluorescence resonance electron transfer (Figure S3D).

### Characterization of NB

An NB was synthesized as the nanocarrier to transport the nanoparticles. TEM images showed the morphology of the NB with the size of 100-300 nm (Figure S4A and S4B). Platinum (Pt) was sputtered on the NB to enhance conductivity for SEM. In the SEM image, the NB exhibited good dispersity and morphology with uniform spherical shape but decomposed in the vacuum environment after sputtering (Figure S4C). The NB was stored in deionized water at 4 °C, which showed a hydrodynamic diameter of 255 nm (Figure S4D).

### Characterization of AuNR@UCNP@NB

**Figure 3:**
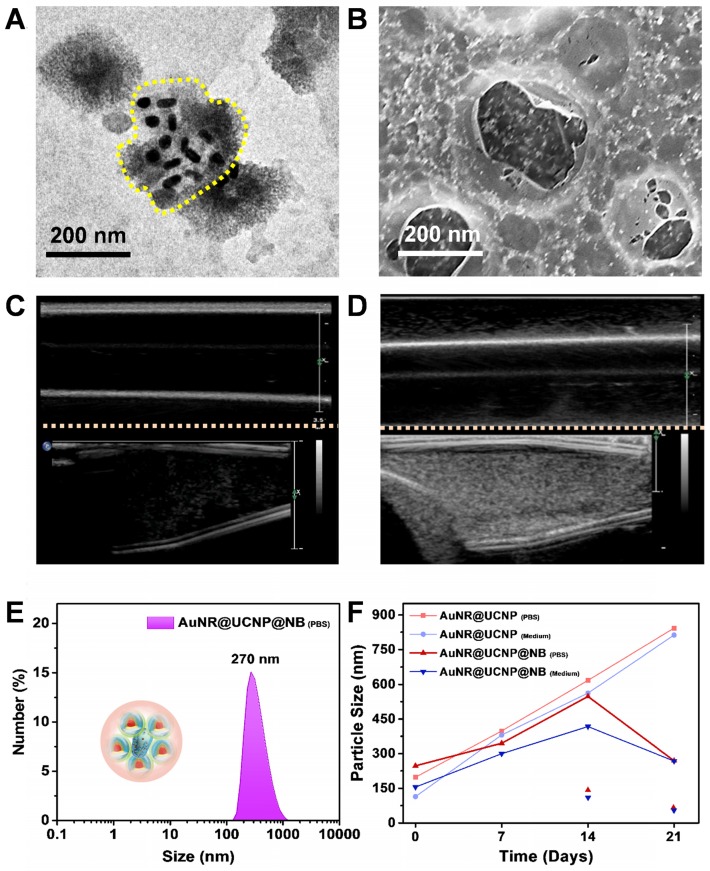
** (A)** TEM and **(B)** SEM images of AuNR@UCNP@NB. Ultrasound imaging of **(C)** water and **(D)** AuNR@UCNP@NB in the Eppendorf. DLS analysis with (E) AuNR@UCNP@NB in PBS and (F) stability of AuNR@UCNP and AuNR@UCNP@NB during 3 weeks of storage in PBS or medium solution.

The upconversion hybrid nanocomposite was encapsulated by the NB. Three types of phospholipids, namely, DPPC, DSPE-PEG2000, and DPPA, were mixed uniformly to synthesize the micelle precursor. The nanocomposite was added before the homogeneous process. Thereafter, the nanosystem with the upconversion hybrid nanocomposite and NB was formed under the octafluoropropane (C_3_F_8_) and nitrogen (N_2_) environment called AuNR@UCNP@NB. During the vacuum environment, the broken NB-embedded with nanocomposite was observed by TEM as shown in Figure 3A. The lipid layer of the broken NB was marked with a yellow curve. The morphology of the NB was evaluated using the Pt sputtering method as previously described. The Pt-coated AuNR@UCNP@NB, which enhanced the conductivity, showed stereoscopic images in Figure 3B. As a contrast agent, the NB also exhibited good distribution in deionized water. The difference after adding the NB was observed in the US images by the Philips iU22 Ultrasound System (Phillip, Amsterdam, Netherland). Figure 3C illustrates that the deionized water without the NB showed dark side image, whereas no US signal can be detected. Thereafter, the increase in the reflective surface of the solution via the addition of NB enhanced the signal and showed white dots, as shown in Figure 3D. The hydrodynamic diameter tested by dynamic light scattering (DLS) showed an average diameter of 270 nm to check the nanosystem maintained in the nanoscale (Figure 3E). Moreover, the stability in the different media was confirmed over 3 weeks. Without the NB embedded, AuNR@UCNP@NB substantially accumulated day by day. The nanosystem showed excellent stability after 3 weeks of storage, which maintained the size at 200-500 nm. After 2 weeks, the AuNR@UCNP can be measured, which indicated that the NB was decomposed (Figure 3F).

### Photothermal and photodynamic effect

**Figure 4:**
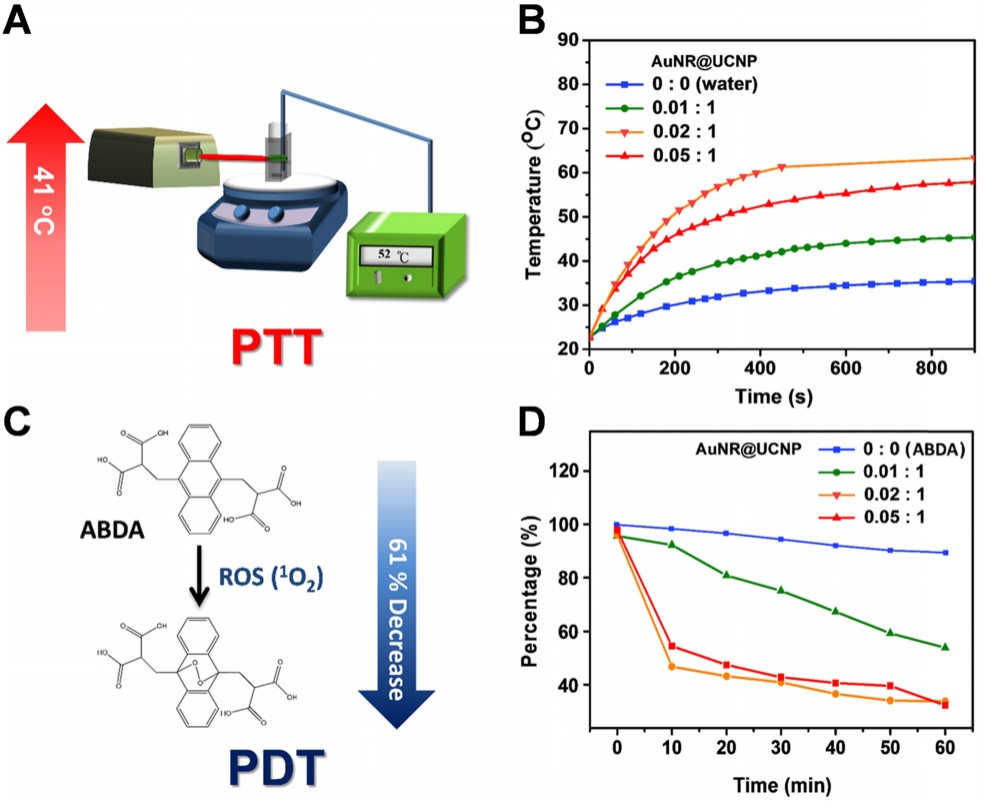
Schematic of the experimental set-up on the **(A)** photothermal (PTT) test and **(C)** photodynamic (PDT) effect. **(B)** Diagram of the thermal change and **(D)** ABDA change with varying concentrations of AuNR@UCNP.

After the basic characterization of the materials, the PTT and PDT effects of the nanosystem were tested sequentially under 808 nm NIR laser. First, the device detected the different ratios between the AuNR@mS and the protamine-coated UCNP for the photothermal effect (Figure 4A). The tin foil was covered around the quartz cuvette with constant stirring to avoid loss of temperature to the environment. Different ratios of AuNR@mS (i.e., 0.01, 0.02, and 0.05 mg and without AuNR@mS) conjugated with 1 mg of protamine-coated UCNP embedded in the NB was detected by the probe of the heater to observe temperature change (Figure 4B). The temperature was measured every 20 s for 15 min. The improved ratio of the photothermal effect was exhibited with 0.02 mg of AuNR@mS and 1 mg of protamine-coated UCNP of the nanosystem. After the 808 nm laser, the temperature of the aqueous solution approached 62 °C with increasing temperature of 41 °C. Second, the PDT effect was measured by the generation of ROS from the photosensitizer MC540. ABDA was used as the ROS marker to detect the ROS concentration in the solution and determine the efficiency of PDT. When the NIR laser triggered MC540, the ROS species were generated and reacted with ABDA. In the first 10 min, the absorption of ABDA rapidly decreased, indicating that many ROS were generated in the solution. The PL spectrum of ABDA measured with 10 min intervals for 1 h decreased because of the photodynamic effect from MC540 (Figure 4C). The improved ratio of the nanosystem (UCNP:AuNR = 1:0.02) also showed a 61% decrease in ABDA (Figure 4D). The considerable PTT and PDT effects were observed in the experiment of the materials. The absorption of AuNR was measured to confirm the photostability under 808 nm laser (1.5 W/cm^2^). Monitor the absorption intensity of 520 nm and 645 nm, the AuNR still presented the constant trend after 60 min laser irradiation confirming the repeatability of PTT (Figure S5).

### Cellular uptake and localization with viability and cytotoxicity

**Figure 5:**
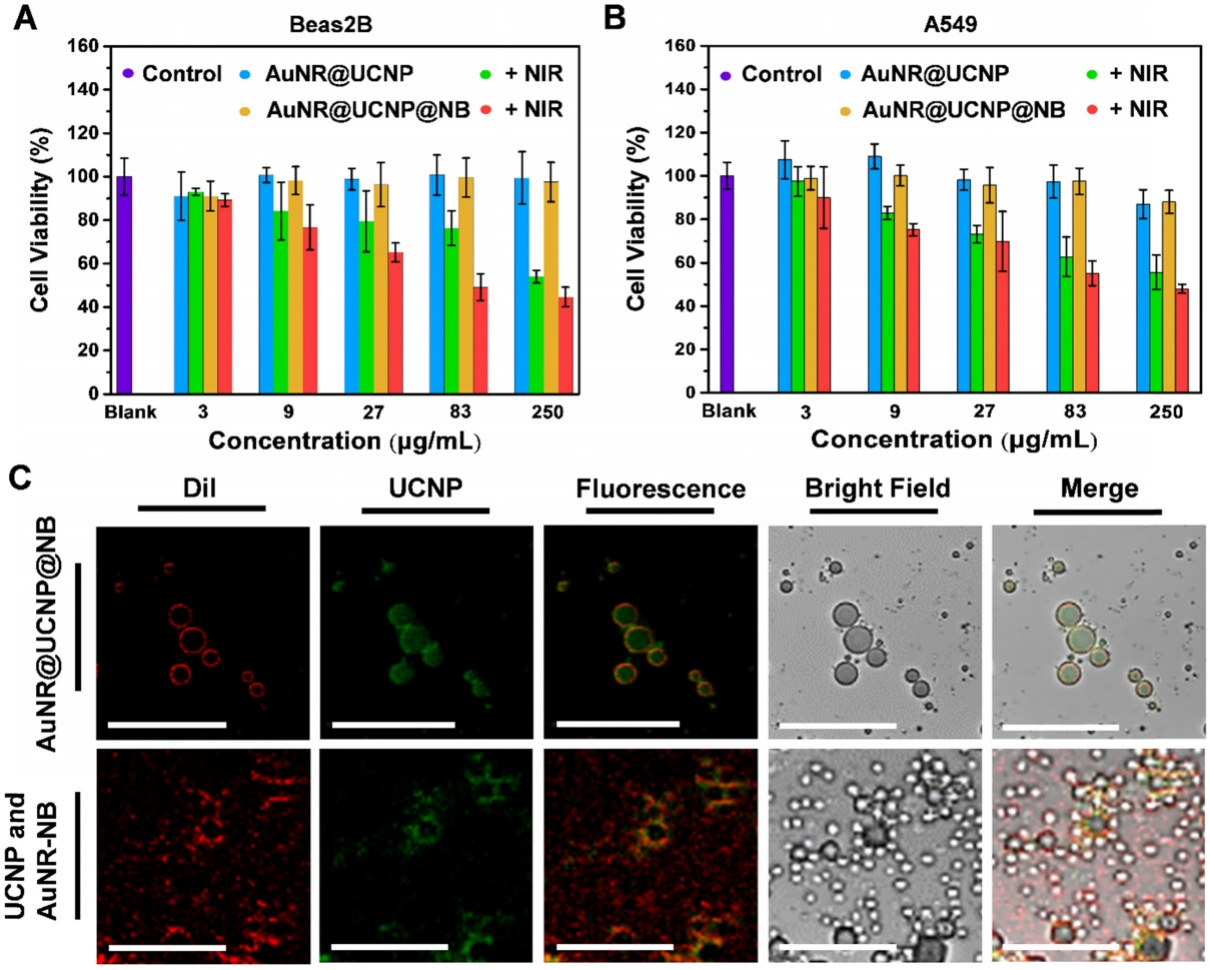
Cytotoxicity treated with AuNR@UCNP@NB with or without 808 nm laser irradiation for **(A)** Beas2B cell line and **(B)** A549 cell line. **(C)** LSCM image of AuNR@UCNP@NB and UCNP with AuNR@NB under LSCM.

Before the biological test, the cytotoxicity of the NIR laser source was confirmed with the different irradiation intensities and times in Figure S6**.** Cell viability was high under those conditions. An *in vitro* test was performed to confirm that the materials also had a good phototherapeutic effect on the organism with low cytotoxicity. The Beas2B normal lung and A549 lung cancer cell lines were selected as the observation objects of the material cytotoxicity. The nanosystem at 3, 9, 27, 81, and 250 μg/mL was added to the individual wells for 48 h and irradiated for a short time. The short-time irradiation of 808 nm laser was used to prevent the medium from overheating (1.5 W/cm^2^, 6 min/well). Figures 5A and 5B show that the toxicity of the nanosystem was only observed under the 808 nm laser. The dual phototherapy (i.e., PTT and PDT) was more cytotoxic than the single phototherapy as the nanosystem concentration was increased. Without the NIR irradiation, the nanosystem was less toxic, thereby showing cell viability of above 80%. The distribution of the nanosystem confirmed the location of the nanocomposite, whether inside the NB, by LSCM. The UCNP inside and outside the NB was used as the experiment and control, respectively. The UCNP nanocomposite was added before (control group) and after (experimental group) the NB film formation to determine whether the material can be coated within the NB. Figure 5C shows that the lipophilic membrane stain DiI was used to mark the NB, the morphology of which can be observed under LSCM. The NB layer emitting the red emission was exhibited with the experiment and control groups. The UCNP distribution with the green emission also showed that the UCNP of the experiment gathered together to form a circle. By contrast, the control showed that the UCNP separated from the NB. When the fluorescence images were merged, the distribution of orange emission showed an overlap between UCNP and NB. With the bright-field images, the NB can be observed directly.

### *In vitro* ROS evaluation with DCFH reagent

The intracellular ROS marker, a membrane-permanent molecule that can pass through the cell membrane, was used to confirm the *in vitro* effect of PDT from the 808 nm-triggered nanosystems. This marker exists in an acetate ester form of 2',7'-dichlorodihydrofluorescein diacetate (H2-DCFDA-AM) before entering the cell. The activity of the marker could be switched on via cellular esterase, turning H2-DCFDA-AM to non-fluorescent H2-DCFDA. The H2-DCFDA disperse inside the cell reacted with ROS to DCF, exhibiting green emission under 480 nm excitation (Figure S7). The results of the intracellular ROS study for the four groups (I: Control; II: UCNP + MC540; III: AuNR@UCNP; IV: AuNR@UCNP@NB) showed that group IV had an obvious signal of green fluorescence after irradiation at 1.5 W/cm^2^ using 808 nm laser for 30 min.

### Apoptosis mechanism of PDT

**Figure 6:**
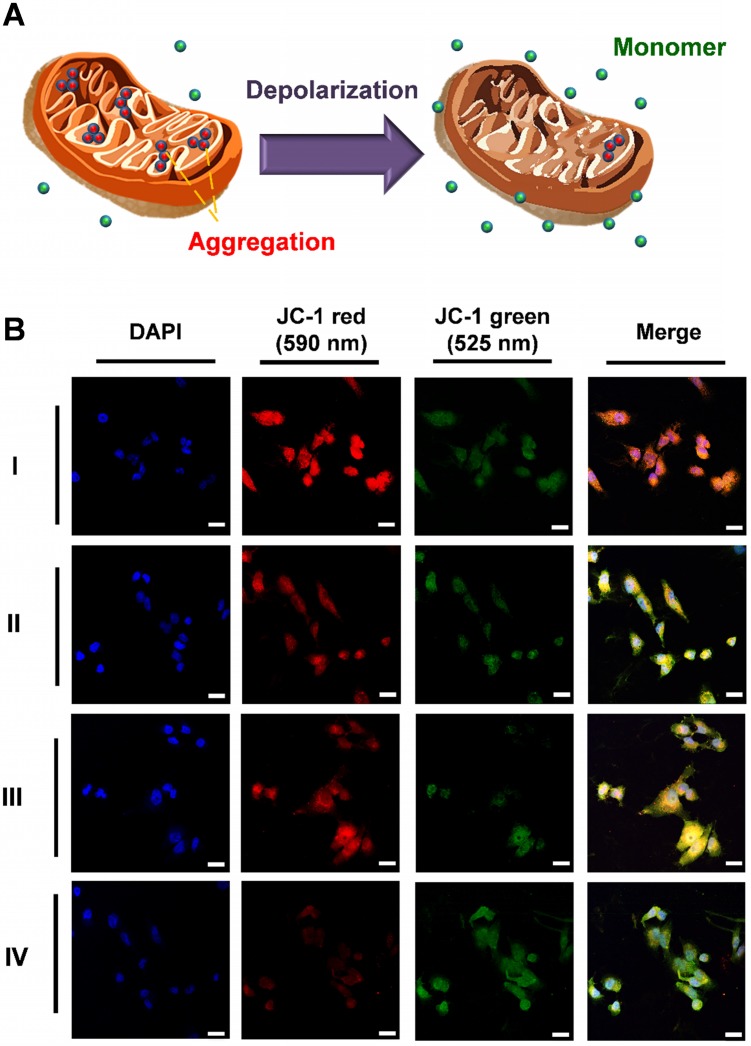
** (A)** JC-1 reagent was applied to test the health of the mitochondria. When the mitochondria worked normal, the JC-1 molecules aggregate and emit red fluorescence. Otherwise, if the mitochondria are damaged, JC-1 is released to the cytosol and the JC-1 monomer molecular emits green fluorescence. **(B)** Fluorescence intensity of the cytometry flow with PE (red emission) and FITC (green emission) filter in four groups (I: Control, II: UCNP + MC540, III: AuNR@UCNP, IV: AuNR@UCNP@NB) with the scale bar = 50 μm.

The ROS produced after PDT treatment interfered with the electron transport chain and was associated with the destruction of the mitochondria. The major pathway of PDT was also apoptotic. JC-1 was used as the stain in the mitochondria to investigate the mechanism underlying the cell apoptotic pathways. The status of the mitochondria was detected on the basis of the change in mitochondrial membrane potential (MMP, ΔΨm), as shown in Figure 6A. Healthy mitochondria exhibit high ΔΨm and negative-potential JC-1 accumulation and gathered in the matrix of the mitochondria passively. After accumulation, the JC-1 polymer showed red emission at 610 nm. Conversely, dead cells with damaged mitochondrion exhibited low ΔΨm because of depolarization. JC-1 separated in the cytoplasm as the monomer with green fluorescence at 570 nm. The A549 cells were subjected to three types of phototherapy with the control group (I: Control; II: UCNP + MC540, PDT; III: AuNR@UCNP, PTT; IV: AuNR@UCNP@NB, dual phototherapy) under the 808 nm laser (1 W/cm^2^ for 5 min). The curable effect of phototherapy was compared in the LSCM images (Figure 6B). The considerable intensity of red emission was observed in the control without the treatment, indicating that the cells were healthy. With the single phototherapy (i.e., II or III), the intensity of the red emission was darker than that of the control. However, the dual therapy (IV) showed the darkest intensity, indicating the best curable effect as well. A series of green emissions showed the opposite trend. The dual therapeutic effect removed JC-1 out of the mitochondrion as monomers. Red signal-like spots (marked with the white arrows) could be observed in the merged LSCM images after single phototherapy. By contrast, the green signals were nearly presented with the dual therapy treatment.

### Flow cytometric analysis and Western blot analysis of apoptosis

**Figure 7:**
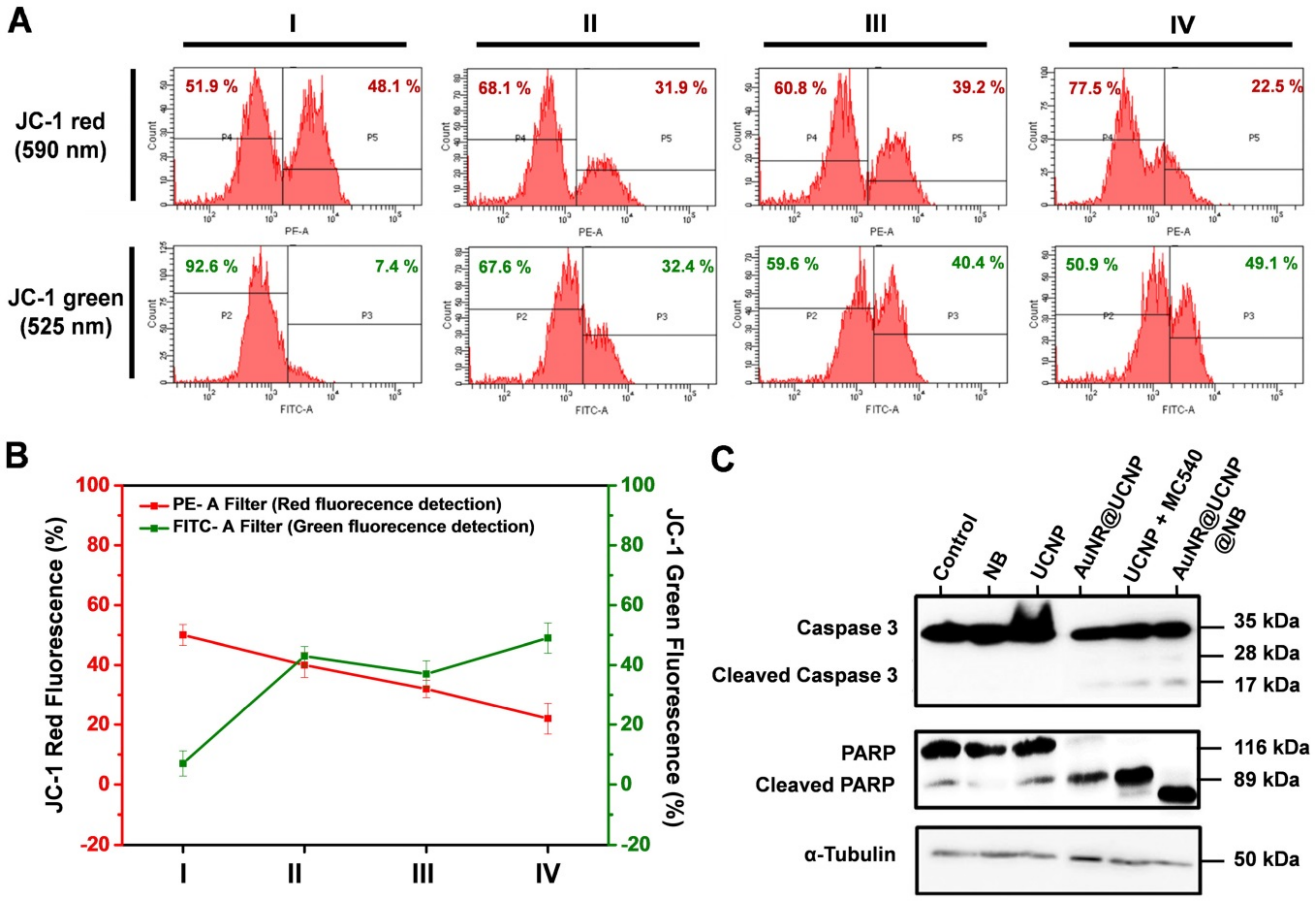
**(A)** Schematic of JC-1 distribution in the mitochondria. **(B)** JC-1 mitochondrial analysis of the A549 cells treated with four groups (I: Control; II: UCNP + MC540; III: AuNR@UCNP, IV: AuNR@UCNP@NB) under LSCM. **(C)** Western blot analysis of the A549 cells with NB, UCNP, AuNR@UCNP, UCNP + MC540, and AuNR@UCNP@NB.

Quantification of the four groups (I: Control; II: UCNP + MC540; III: AuNR@UCNP; IV: AuNR@UCNP@NB) by flow cytometry confirmed the red and green fluorescence of JC-1 (Figure 7A). Specific filters were used to collect the different wavelengths of fluorescence. To detect the green and red fluorescence from JC-1, we chose FITC (green) and PE (red) filters under flow cytometry. The majority of the cells exhibited autofluorescence with the intensity around 10^3^, as in P2 and P4. The P5 area showed the intensity of the JC-1 red emission. After the dual therapeutic effect (group IV), the red intensity declined from 48.1% to 22.5%, indicating that this effect performed better than the photodynamic effect (group II, 31.9%) and the photothermal effect (group III, 39.2%). The trend of the green emission of JC-1 also matched the LSCM images. The more curable therapy was used, the green emission from JC-1 monomer showed higher. This experiment was repeated three times as shown in Figure 7B to produce reliable quantitative data. Caspase 3 and PARP as the apoptosis markers were chosen to track the apoptotic pathway, which can be confirmed by Western blot analysis. Figure 7C shows that NB, UCNP was regarded as the control group. The experimental groups under the irradiation treatment were AuNR@UCNP, UCNP + MC540, and AuNR@UCNP@NB. The high expression of the cleaved forms of caspase 3 (17 and 28 kDa) and PARP (89 kDa) was revealed with the source of the phototherapeutic effect (AuNR or MC540). The preceding results confirmed that the A549 cells were induced to death through the apoptotic pathway from the toxic ROS to destroy the mitochondria.

**Figure 8:**
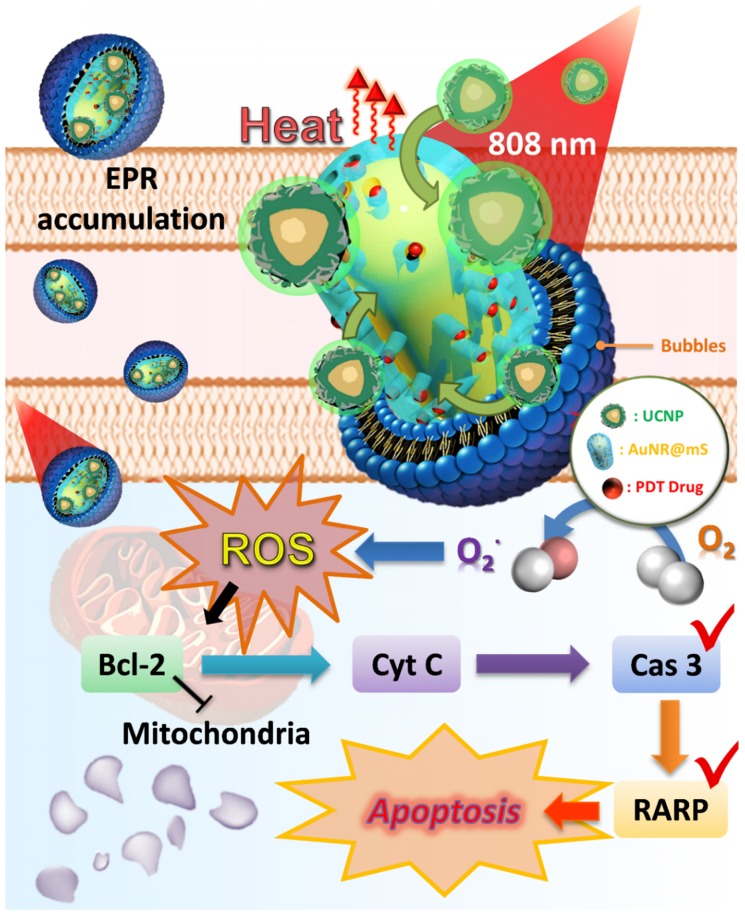
Schematic of the dual phototherapy on the mitochondria with the mechanism of cell death.

The *in vitro* test indicated that the pathway of the dual-model phototherapy was reviewed as shown in Figure 8. The NB was an efficient nanocarrier to transport AuNR@UCNP and accumulated passively through the EPR effect in cancer cells via endocytosis. The nanocomposite was released after the US treatment. The theranostic platform supplied PTT and PDT under the 808 nm laser source. The increase in temperature from the AuNR resulted in the different patterns of cell death, such as apoptosis, necroptosis, and necrosis. The dominant pattern of necrosis was presented at a high temperature. Under irradiation, the photosensitizer MC540 also released and produced ROS, thereby inhibiting the expression of Bcl2. Therefore, cytochrome c was induced indirectly for the initiation of apoptosis. The overexpression of cleaved caspase 3 and PARP also confirmed the apoptotic pathway of the mitochondria with the PDT treatment.

### *In vivo* dual-model imaging and phototherapeutic effect

**Figure 9:**
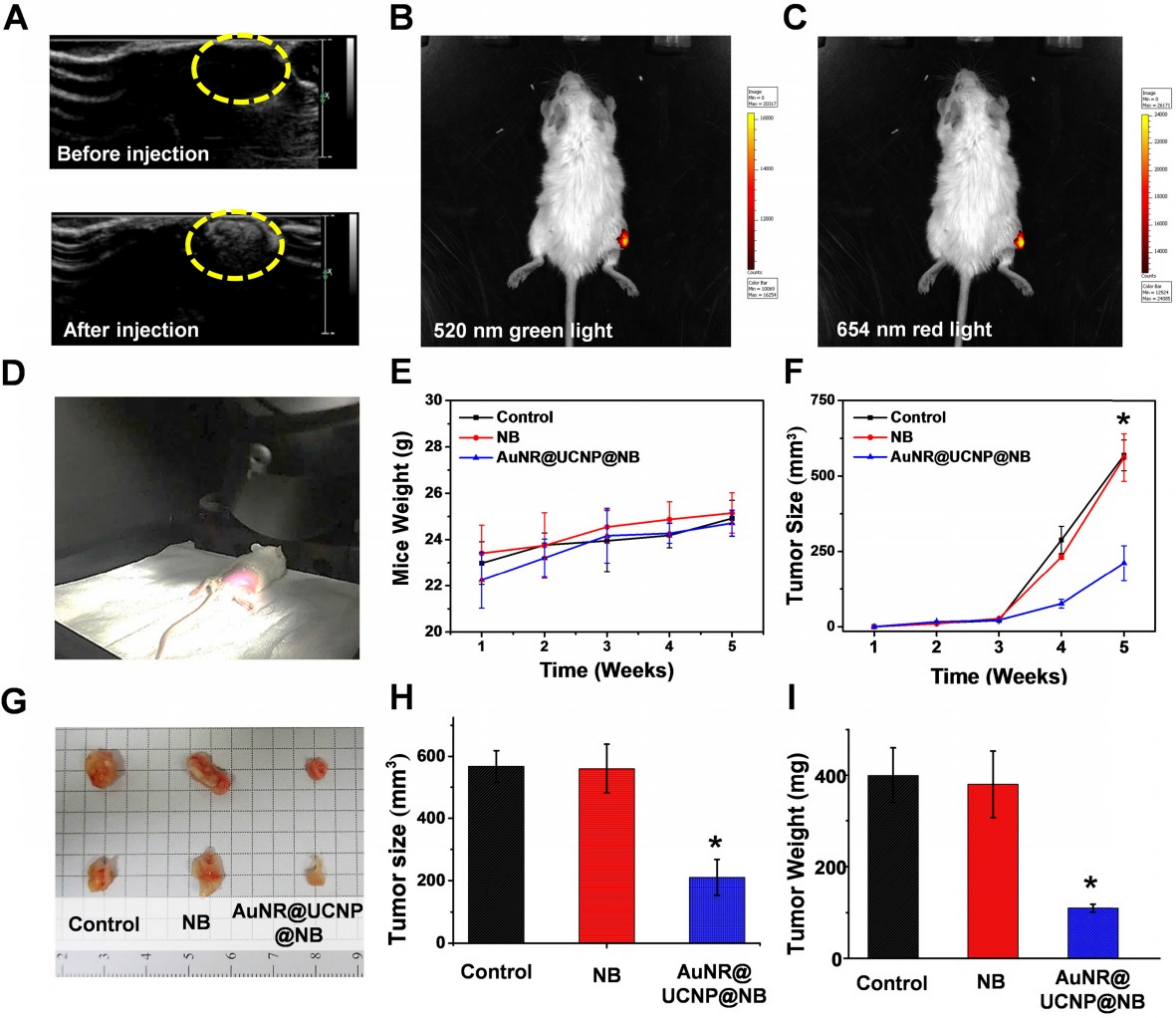
*In vivo* diagnostic results show that AuNR@UCNP@NB can generate the **(A)** ultrasonic signal after injection. The UCNP fluorescence can also be detected at the major **(B)** 520 nm and** (C)** 654 nm emission. **(D)** Set-up for the dual phototherapy treatment *in vivo* test and **(G)** image of the tumor after the treatment. Diagrams of the **(E)** mouse weight and **(F)** tumor size compared NB and AuNR@UCNP@NB during the treatment for 5 weeks. After the treatment, **(H)** relative tumor size, and **(I)** tumor weight of the groups of NB and AuNR@UCNP@NB with the control group.

**Figure 10:**
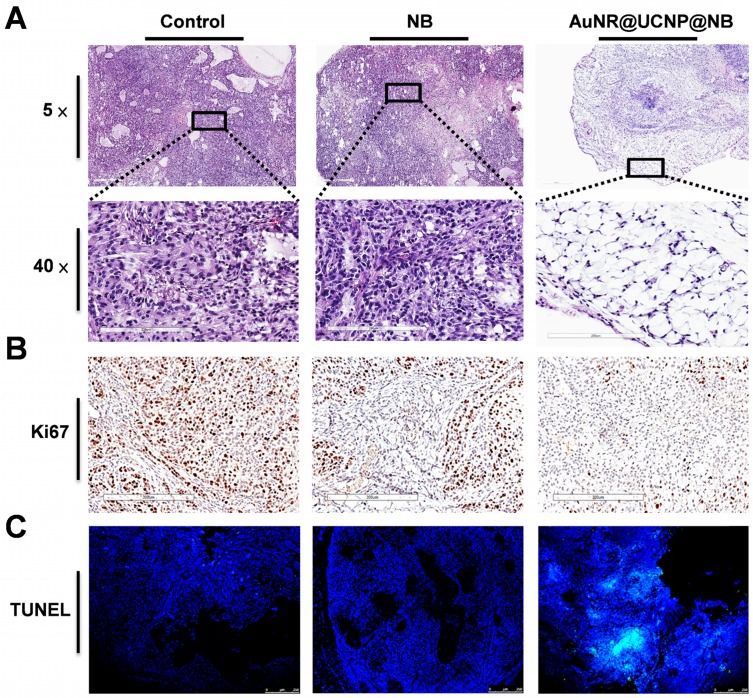
A549 tumor tissue staining. **(A)** 5× and 40× H&E, **(B)** immunohistochemistry, and **(C)** TUNEL tissue staining to present the change among the “Control,” “NB” only, and “AuNR@UCNP@NB” groups. The Ki67 stain reveals the brown spots overlapping with the cell nucleus. TUNEL-FITC is overlapped with DAPI as observed by LSCM.

This study developed a multiple imaging and treatment nanosystem. In the AuNR@UCNP@NB material, the upconverted fluorescent signal was provided by the UCNP and the US signal was provided by the NB, which allowed the system to achieve high-resolution and high-spatiality diagnostic imaging. In Figure 9A, US images before and after applying AuNR@UCNP@NB confirmed that the NB can be used as a good contrast agent in mice and effectively visualize the shape of the tumor. However, the US imaging cannot exactly locate the position of the material because the resolution of the US signal is insufficient to obtain the corresponding data. Therefore, the fluorescent images provided by high-resolution UCNP are important. In Figures 9B and 9C, the tumor on the right thigh of the mouse accumulated AuNR@UCNP@NB, which can be measured by the IVIS-spectrum Xenogen system (PerkinElmer, MA, USA) to observe the upconverted green and red lights at 520 nm and 654 nm, respectively. For multiple phototherapies, Figure 9D shows the real situation of the dual-model phototherapy in the *in vivo* test. The two groups were separated as NB and AuNR@UCNP@NB and chosen as the experimental groups. A549 lung cancer cells were planted in each mouse via subcutaneous injection in the first week and achieved the size of 560 mm^3^ under observation and weekly inspection. The signal of US imaging was observed immediately and the flow of the nanoparticles was tracked with fluorescent imaging. The tumors were treated with the 808 nm laser source (500 mW/cm^2^ for 30 min) during the fourth and fifth weeks. The mouse weight was measured to check their health as shown in Figure 9E. Although no evident difference was observed in the weight, the slight trend can only be observed in the AuNR@UCNP@NB of the experimental group, whereas all the other groups exhibited an increasing trend. The results indicated that dual therapy caused light-burden with a low side effect on the mouse. After the irradiation treatment for 2 weeks, the curable effect was reflected in the tumor size in each group (Figure 9F). The tumor size of the group without the curable effect (control and NB) was approximately 567 mm^3^. Compared with the other experimental groups (NB and AuNR@UCNP@NB), the dual-model phototherapy (AuNR@UCNP@NB) considerably inhibited the tumor size (approximately 220 mm^3^) and presented a better curable effect. The tumor size in the group treated with the NB was similar to that in the control group. Given its nontoxicity, the NB has great potential in medical applications. The mouse was sacrificed on the fifth week, and the tumor was removed as shown in Figure 9G. The improved dual-model phototherapy reduced the tumor size and weight by almost one fold (Figures 9H and 9I). After the sacrifice, the tumor tissue was collected via H&E staining. Expectably, the removed tumors in the groups of AuNR@UCNP@NB showed evident damage (Figure 10A). The result of H&E staining showed that the distribution of tumor cells became hollow and sparse after treatment with AuNR@UCNP@NB. Staining results showed that the growth of some of the cells was blocked by oxidative stress. The other cells directly became voids because of thermal damage (Figure S8). Moreover, we performed Ki67 IHC and TUNEL tissue staining to confirm the proliferation and apoptosis of the cells. Ki67 was used as a marker to discriminate the degree of malignancy of the tumor and assess the prognosis and predictive value in the diagnosis and treatment of cancer. The control group had a higher performance of Ki67 at IHC resolution than the AuNR@UCNP@NB group, which exhibited a low expression level of Ki67 because of its inhibitory effect on cancer cells (Figure 10B). Conversely, TUNEL detected DNA fragments generated by a cascade of apoptotic signals, and the nicks present on DNA can be used to label cells that are severely damaged. Therefore, AuNR@UCNP@NB has a strong TUNEL green fluorescent light (Figure 10C). This result shows that AuNR@UCNP@NB can effectively achieve optimal tumor suppression by photothermal and photodynamic dual light therapy.

## Conclusions

In this study, we developed and showed the therapeutic functions of the novel nanosystem with the combination of multiple theranostic nanoplatforms that can be triggered and activated by 808 nm NIR laser and US. The nanocomposite was assembled with AuNR@mS and UCNP via protamine, which was encapsulated in NB. The complementary multi-imaging solved the poor US resolution from NB and insufficient penetration depth of fluorescence by the combination of ultrasonic diagnosis and fluorescence imaging. LSCM results exhibited that the nanosystem showed good accumulation and distribution in the cancer cells. However, dual phototherapy was also comparable with the traditional single phototherapy. The dual phototherapeutic effect from AuNR (PTT) and MC540 (PDT) increased considerably, thereby inducing cancer cell death with the mechanism confirmation. The apoptotic pathway of the mitochondrion was explored further via JC-1 staining. The results of *in vitro* and *in vivo* analyses confirmed the curative effect of improved dual phototherapy.

## Abbreviations

AuNR: gold nanorod
AuNR@mS: mesoporous silica-coated AuNR
MC540: merocyanine 540
UCNP: upconversion nanoparticle
AuNR@UCNP: AuNR@mS conjugated with 808 nm UCNP nanocomposite
NB: nanobubble
AuNR@UCNP@NB: AuNR@UCNP embedded in the NB nanosystem
PTT: photothermal therapy
PDT: photodynamic therapy
ROS: reactive oxygen species
EPR: enhanced permeability and retention
