# Supplementary material for: Theranostic nanobubble encapsulating a plasmon-enhanced upconversion hybrid nanosystem for cancer therapy
Source: Theranostics. 2020 Jan 1;10(2):782–96. doi: 10.7150/thno.38684 (PMC6929987; doi:10.7150/thno.38684)
Supplement: Supplementary file 1 — Supplementary figures and tables. [file thnov10p0782s1.pdf]

Supplementary Material

Theranostic nanobubble encapsulating a plasmon-enhanced upconversion hybrid nanosystem for cancer therapy

Wen-Tse Huang[‡,1,2], Ming-Hsien Chan[‡,3], Xueyuan Chen[2], Michael Hsiao[*,3,4], Ru-Shi Liu[*,1,3,5]

1. Department of Chemistry, National Taiwan University, Taipei 106 Taiwan
2. CAS Key Laboratory of Design and Assembly of Functional Nanostructures, and Fujian Key Laboratory of Nanomaterials, Fujian Institute of Research on the Structure of Matter, Chinese Academy of Sciences, Fuzhou, Fujian 350002, China
3. Genomics Research Center, Academia Sinica, Taipei 115 Taiwan
4. Department of Biochemistry College of Medicine, Kaohsiung Medical University, Kaohsiung, 807 Taiwan
5. Department of Mechanical Engineering and Graduate, Institute of Manufacturing Technology, National Taipei University of Technology, Taipei, 106 Taiwan

*Correspondence to:

Dr. Ru-Shi Liu, E-mail: rsliu@ntu.edu.tw, or to:

Dr. Michael Hsiao, E-mail: mhsiao@gate.sinica.edu.tw

Materials

All chemicals were used without purification. Hexadecyltrimethylammonium bromide ($C_{19}H_{42}BrN$, 99.0%; CTAB), gold (III) chloride trihydrate ($HAuCl_4 \cdot 3H_2O$, 99.9%), sodium borohydride ($NaBH_4$, 98%), sodium salicylate ($NaC_7H_5O_3$, 99.5%), silver nitrate ($AgNO_3$, 99%), L-ascorbic acid ($HC_6H_7O_6$, 99%), tetraethyl orthosilicate ($Si(OC_2H_5)_4$, 98%), hydrochloric acid (HCl, 36.4%), and merocyanine 540 (MC540, 90%) were purchased from Sigma-Aldrich and used in the AuNR synthesis. The yttrium, ytterbium, erbium, holmium, and neodymium of rare-earth hydrate acetate ($N(CH_3CO_2)_3$ H_2O, N = Y, Yb, Er, Ho, Nd), oleic acid ($C_{18}H_{34}O_2$, 99%; OA), 1-octadecane ($C_{18}H_{36}$, 99.5%; ODE), sodium hydroxide (NaOH, 98%), ammonium fluoride (NH4F, 98%), cyclohexane (C_6H_{12}, 99.5%), ethanol (CH_3CH_2OH, ≥99.8%), and protamine were used in the UCNP synthesis and acquired from Sigma-Aldrich. Three types of phospholipids from Avanti Polar Lipids, namely, 1,2-distearoyl-sn-glycero-3-phosphoethanolamine-N-[methoxy(polyethyleneglycol)-2000] (DSPE-PEG2000), 1,2-dipalmitoyl-sn-glycero-3-phosphocholine (DPPC), and 1,2-dihexadecanoyl-sn-glycero-3-phosphate (DPPA), were used in the NB synthesis. Chloroform ($CHCl_3$), glycerol ($C_3H_8O_3$), and pluronic F-127 were acquired from Midland Scientific, J.T. Baker, and Sigma-Aldrich, respectively, which were also used in the NB synthesis. The staining agents and analytical chemicals, including 1,1'-dioctadecyl-3,3,3',3'-tetramethylindocarbocyanine perchlorate ($C_{59}H_{97}ClN_2O_4$, 98%; DiI), 9,10-Anthracenediyl-bis(methylene)dimalonic acid ($C_{22}H_{18}O_8$, 90%; ABDA), and 2′,7′-dichlorodihydrofluorescein diacetate ($DCFH_2$-DA), were purchased from Sigma-Aldrich. The biological analytic chemicals, such as 4',6-diamidino-2-phenylindole ($C_{16}H_{15}N_5$; DAPI) and 5,5',6,6'-tetrachloro-1,1',3,3'-tetraethylbenzimidazolylcarbocyanine iodide ($C_{25}H_{27}Cl_4IN_4$; JC-1), were acquired from Thermo Fisher Scientific. The Keratinocyte-SFM and F-12K media were

purchased from Thermo Fisher Scientific for seeding the Beas2B normal lung bronchus and A549 lung cancer cells, respectively. That is, the penicillin–streptomycin–glutamine (PSG) supplement for culture media was purchased from GIBCO.

Supplementary characterization

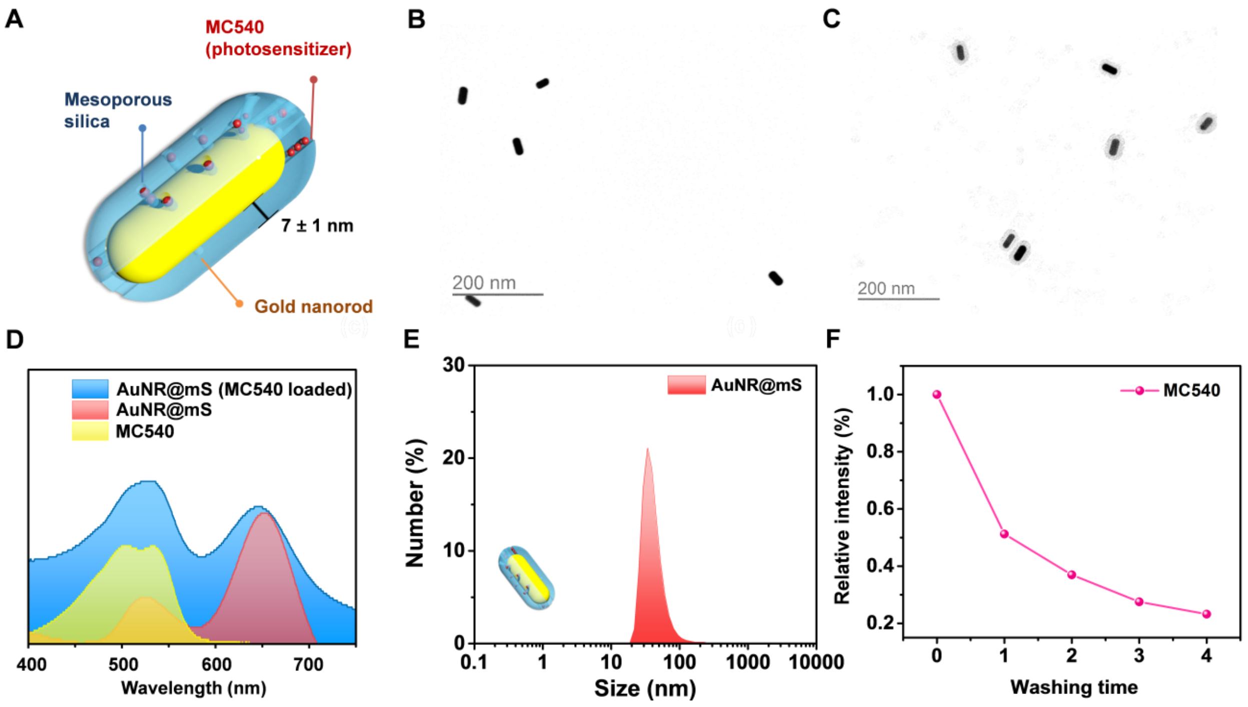

Figure S1. (A) Schematic of AuNR@mS (MC540 loaded). TEM images of (B) AuNR and (C) AuNR@mS. (D) Absorbance spectrum of AuNR, MC540, and AuNR@mS-MC. (E) DLS analysis of AuNR@mS-MC. (F) Loading diagram of photosensitizer, MC540 with centrifugations and washed four times to ensure that MC540 was loaded in AuNR@mS.

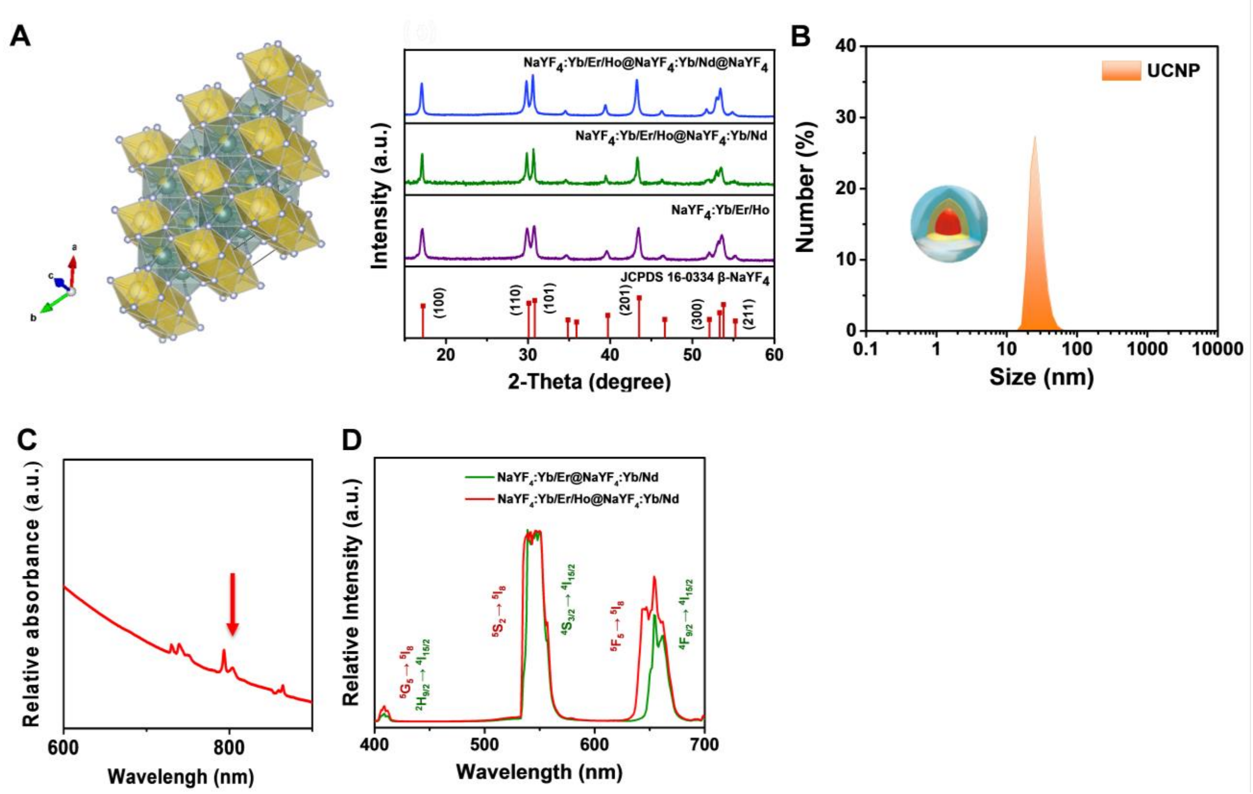

Figure S2. (A) X-ray diffraction patterns of upconversion nanoparticle $NaYF_4$:Yb/Er/Ho (core), $NaYF_4$:Yb/Er/Ho@$NaYF_4$:Yb/Nd (core-shell), and $NaYF_4$:Yb/Er/Ho@$NaYF_4$:Yb/Nd@$NaYF_4$ (core-shell-shell) with the crystal structure of the hexagonal phase. (B) The hydrodynamic diameter of UCNP. (C) Absorbance spectrum of UCNP and (D) PL spectrum of different doping in the core, $NaYF_4$:Yb/Er@$NaYF_4$:Yb/Nd and $NaYF_4$:Yb/Er/Ho@$NaYF_4$:Yb/Nd.

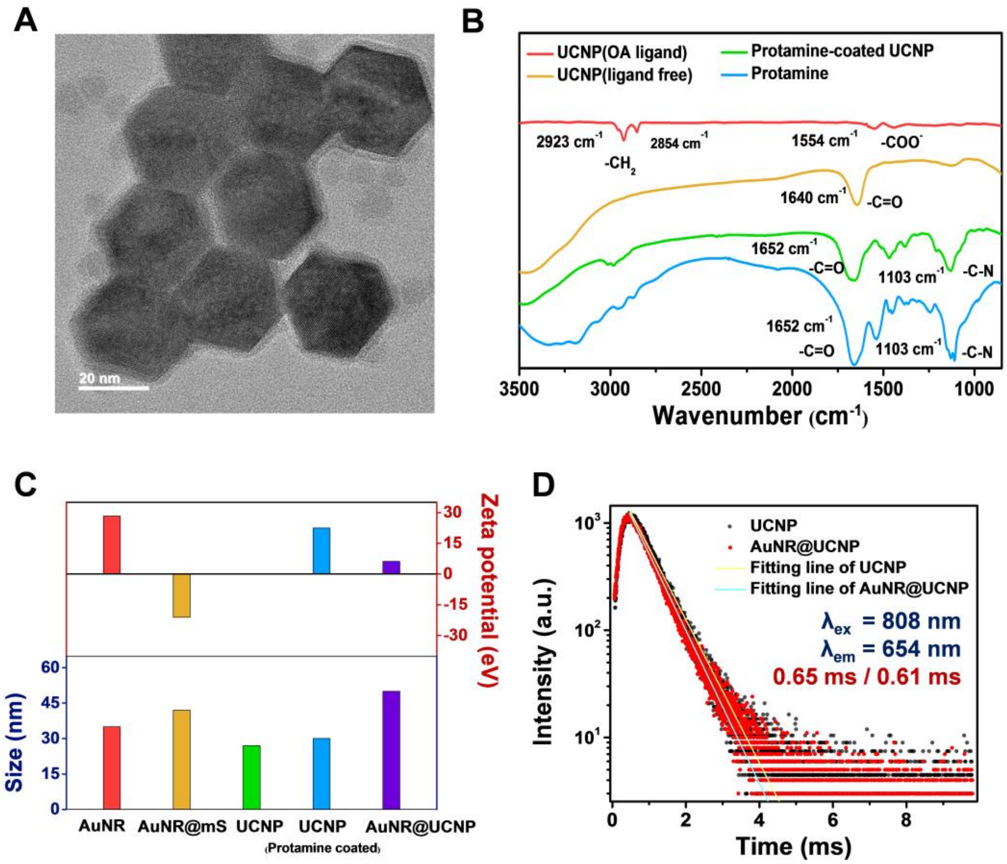

Figure S3. Protamine-coated UCNP with (A) TEM images and (B) FTIR spectrum. (C) Zeta potential and hydrodynamic diameter of each nanomaterial. (D) Lifetime measurement of UCNP and AuNR@UCNP.

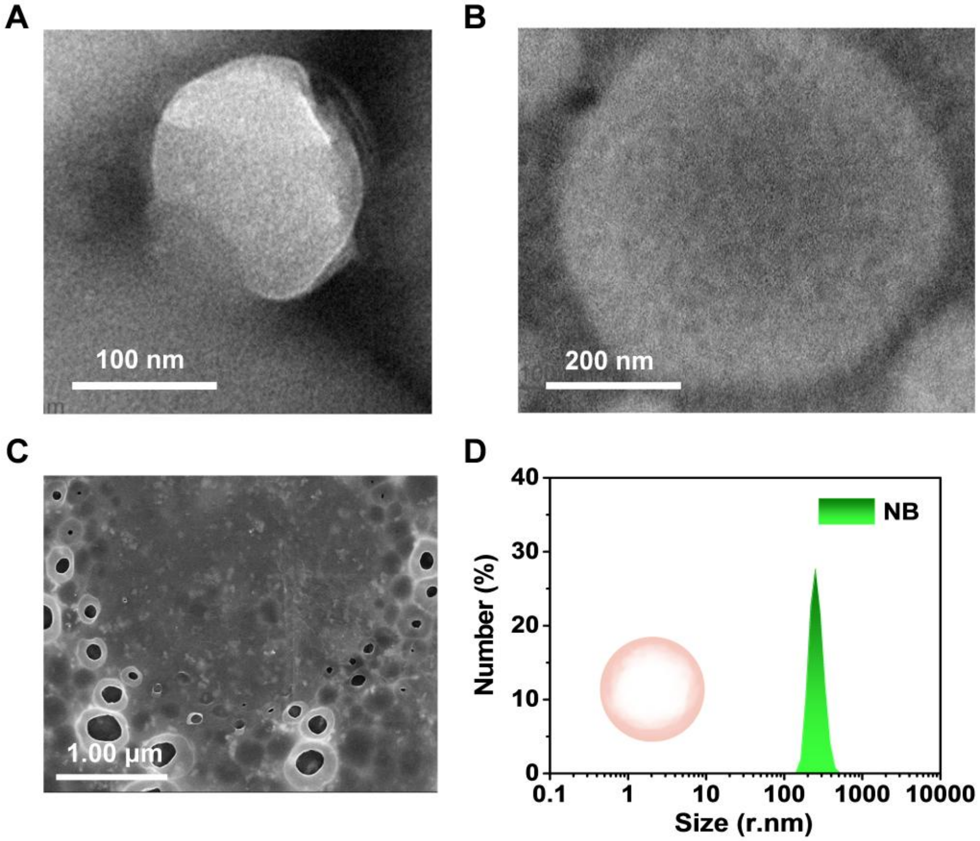

Figure S4. TEM images of NB at approximately (A) 100 nm and (B) 200 nm. (C) SEM image and (D) DLS analysis of NB.

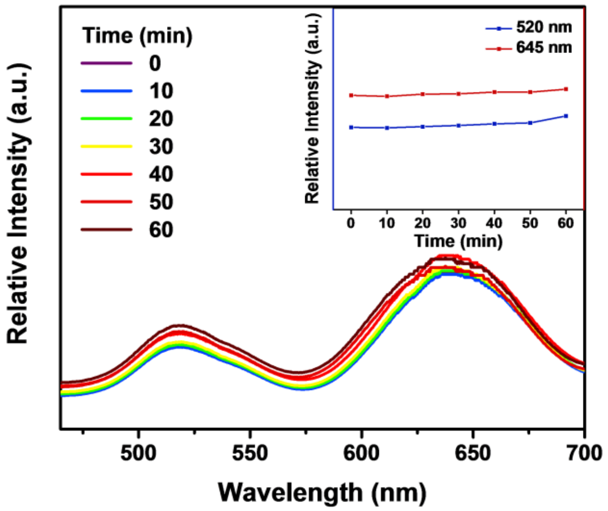

Figure S5. The absorption spectrum of AuNR under 808 nm laser irradiation for an hour (monitoring at 520 nm and 645 nm).

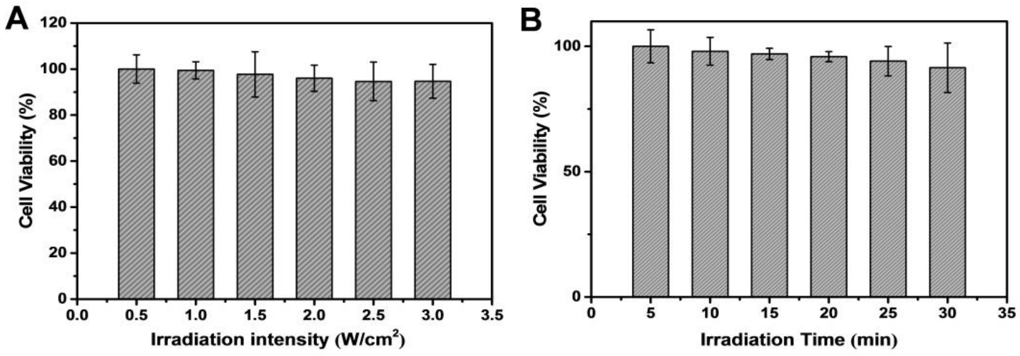

Figure S6. (A) Different intensities and (B) times of 808 nm NIR laser irradiation conditions were used to treat Beas2B normal cells. Results show that the different treatments of 808 nm light irradiation do incur obvious damage to the cells.

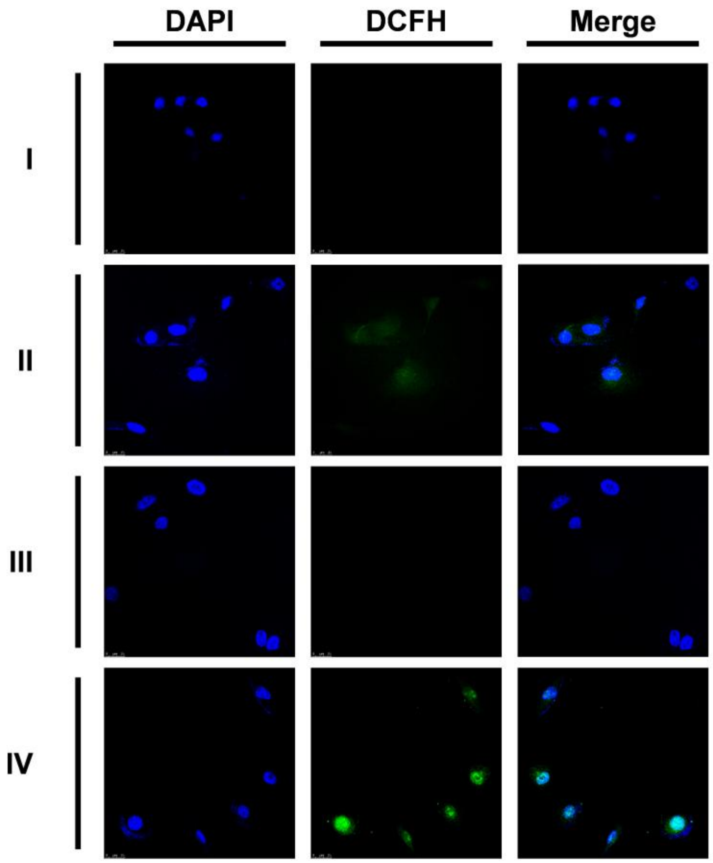

Figure S7. ROS evaluation of A549 cells incubated with four groups (I: Control, II: UCNP + MC540, III: AuNR@UCNP, IV: AuNR@UCNP@NB) by staining with DCF could be detected by LSCM at 550 nm.

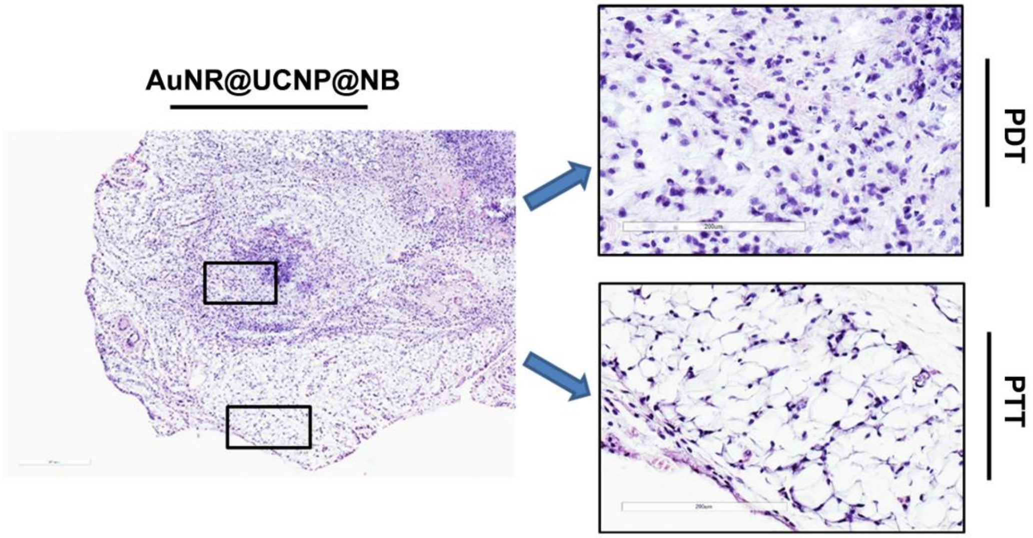

Figure S8. H&E tissue staining of the AuNR@UCNP@NB group and the entire tumor tissue is treated in two different ways (PDT + PTT). The PDT oxidative stress causes a decrease in cell density in the center of the tumor growth and the PTT heat treats more peripheral cells to produce the tissue cavity, respectively.
